# Chromatin and higher-order chromosome organization shape the recombination landscape in *C. elegans*

**DOI:** 10.1186/1756-8935-6-S1-O17

**Published:** 2013-03-18

**Authors:** Chitra V Kotwaliwale, Sasha A Langley, Andrea C Dose, Clara W Wang, Abby F Dernburg

**Affiliations:** 1 Department of Molecular and Cell Biology, University of California, Berkeley, CA 94720, USA; 2 Howard Hughes Medical Institute, Chevy Chase, MD 20815, USA; 3 Department of Genome Dynamics, Life Sciences Division, Lawrence Berkeley National Laboratory, Berkeley, CA 94720, USA

## 

Meiotic recombination generates crossovers, which physically link homologous chromosomes, and direct their segregation to opposite poles. Despite the benefits, crossovers are restricted both in number as well as spatially. Crossovers in the nematode *C. elegans* occur most frequently within the distal regions (or “arms”) of the chromosomes, which comprise ~50% of the genome, but ~90% of the genetic map length. Meiotic recombination initiates with the formation of programmed double strand breaks (DSBs) catalyzed by the SPO-11 enzyme. It has been unclear whether the crossover bias in *C. elegans* is a consequence of DSB distribution, or instead reflects a mechanism that biases the downstream repair outcome of DSBs. In order to address this, we generated a genome-wide map of meiotic DSBs by mapping the distribution of the sole *C. elegans* RecA-like recombinase, RAD-51, by ChIP-seq. We have found that the RAD-51 binding pattern is strikingly similar to the known recombination pattern in *C. elegans.* The majority of RAD-51 binding sites occur on chromosome arms. Comparing RAD-51 distribution relative to functional genomic elements revealed that RAD-51 is particularly enriched in intronic DNA while coding sequences are underrepresented in RAD-51 occupied regions. We find that monomethylation of histone H3 lysine 36 (H3K36me1), which has not previously been associated with meiotic recombination, is highly correlated with break site preference. H3K36me1 is particularly enriched over active genes with high intron content, suggesting that the specific chromatin environment associated with these genes favors DSB formation in *C. elegans.* We have also identified a sequence motif that is highly overrepresented in DSB sites. Although the DSB pattern is highly correlated with crossover frequency, there are interesting exceptions. In particular, the sub-telomeric regions are completely devoid of crossovers but are active DSB sites. We have also identified a DNA sequence motif that appears to be enriched within DSB sites suggesting that sequence specific factors may have a role in DSB specification in *C. elegans*.

In a parallel study, we have developed an Illumina Golden Gate SNP array to rapidly measure genome-wide recombination rates in *C. elegans*. Using this array we measured meiotic recombination rates in *met-2*(*n4256*) mutant animals. MET-2 is one of the two histone methyltransferases responsible for H3K9 methylation, a histone mark associated with heterochromatin. We find that the genetic map is significantly altered in *met-2*(*n4256*) mutant animals. In particular, regions that are normally enriched for H3K9 methylation show elevated recombination rates in *met-2*(*n4256*) mutants. We are currently investigating whether this change in the genetic map is the result of altered DSB distribution. Taken together, our data provide new insights into the role of chromatin in meiotic recombination. Because meiotic recombination generates genetic diversity, our data implicates chromatin as a key player in the evolution of genomes.

